# Exosome-transmitted LUCAT1 promotes stemness transformation and chemoresistance in bladder cancer by binding to IGF2BP2

**DOI:** 10.1186/s13046-025-03330-w

**Published:** 2025-03-03

**Authors:** Yonghao Zhan, Zhenzhen Zhou, Zhaowei Zhu, Lianghao Zhang, Shuanbao Yu, Yuchen Liu, Xuepei Zhang

**Affiliations:** 1 Department of Urology, The First Affiliated Hospital of Zhengzhou University, Zhengzhou, 450003 China; 2 Shenzhen Institute of Translational Medicine, Health Science Center, Shenzhen Second People’s Hospital, The First Affiliated Hospital of Shenzhen University, Shenzhen, Guangdong China

**Keywords:** Bladder cancer, Cancer stem cell, Exosome, Organoid, Chemoresistance

## Abstract

The chemotherapy resistance is an awkward challenge in management of bladder cancer (BC). Cancer organoid model is an effective preclinical tumor model that could faithfully represent clinical manifestations and simulate the biological processes of chemoresistance. Recent studies have revealed that cancer stem cells (CSCs) play a significant role in the development of chemoresistance in cancer. Exosomes act as essential intercellular messengers and participate in controlling the conversion of distinct cell characteristics, including chemoresistance. However, the role of exosome-transmitted lncRNAs in bladder cancer chemoresistance has rarely been reported. In this study, cancer organoid models were developed from urothelial carcinomas to explore the pathophysiology mechanism of BC chemoresistance, and RNA-seq was performed to screen for lncRNAs involved in chemoresistance of BC. We found chemotherapy enriches stem-like cells in BC, and significant upregulation of Lung Cancer Associated Transcript 1 (LUCAT1) occurs in chemotherapy-resistant organoids and correlated with chemotherapy response. Further experimental results demonstrated that LUCAT1 promotes chemoresistance in bladder cancer by enhancing the stemness phenotype of BC cells in vivo and in vitro. Moreover, exosomes derived from bladder cancer stem cells can enhance the stemness phenotype and chemoresistance of BC cells by delivering LUCAT1. Mechanistically, LUCAT1 could significantly enhance the mRNA stability of HMGA1 via binding to IGF2BP2 in an m6A-dependent manner. The study demonstrates an important role for exosome-transmitted LUCAT1 in chemoresistance and LUCAT1 has the potential to function as both a diagnostic biomarker and therapeutic target for BC.

**Supplementary Information:**

The online version contains supplementary material available at 10.1186/s13046-025-03330-w.

## Introduction

Bladder cancer (BC) has increased significantly in incidence and mortality over the past decade [1–3], among the most common malignancies in the world and the most common genitourinary malignancy in men [4, 5]. For the past decade, Gemcitabine (GEM)-based chemotherapy has been employed as the first-line chemotherapy regimen for advanced UC, yet its effectiveness is suboptimal because the emergence of resistance after a prolonged period of utilization [6–8]. There are several factors that can contribute to the development of resistance to chemotherapy drugs in bladder cancer cells, including accumulation of genetic, epigenetic changes, activation of drug efflux transporters, alterations in drug metabolism, DNA damage repair, activation of cellular survival pathways, and the tumor microenvironment [9–13]. Additionally, the presence of cancer stem cells, which possess self-renewal and differentiation capabilities, has been implicated in chemotherapy resistance [14–17]. Therefore, exploring the molecular mechanism of chemotherapy resistance (CR) and identifying effective prognostic markers capable of predicting bladder cancer outcomes has become increasingly important in order to improve clinical strategies.

Exosomes have a diameter of 30–120 nm and a lipoid bilayer that are released by different types of cells and are absorbed by neighboring or distant cells, creating advantageous positive feedback circuits in cellular communication [18–20]. The main operation of exosomes is to mediate cell-cell interaction through the exchange of proteins, genetic material, miRNAs and lncRNAs [21–24]. Recent research has suggested that exosomes are believed to influence biological functions of both healthy and cancerous cells, as well as influencing tumor immunoregulation, angiogenesis, invasion and metastasis [25–28]. These findings hold important implications for the field of cancer therapy, indicating that these vesicles may be utilized to facilitate targeted drug delivery and personalized treatment options for patients [29–31]. Recent studies have provided evidence that exosome-mediated transfer of lncRNAs from cancer stem cell (CSC) can modify the biology of chemotherapy sensitive (CS) cancer cells, thus potentially facilitating drug resistance [32–34]. Thus, we postulated that exosomes could possibly be implicated in chemoresistance of bladder cancer by mediating the transfer of lncRNAs.

Long non-coding RNAs (lncRNAs) have emerged as crucial regulators of cellular processes and have been implicated in various aspects of cancer biology, including chemotherapy resistance [35–37]. In the context of cancer treatment, substantial evidence has demonstrated the critical involvement of lncRNAs in the regulation of cellular pathways that promote tumor cell survival and confer chemotherapy resistance [38]. These pathways encompass crucial cellular processes such as apoptosis, DNA damage response, and drug efflux [39–41]. Moreover, lncRNAs have been established as key regulators of cancer stem cells, which significantly contribute to the development of chemotherapy resistance [42–44]. Furthermore, the influence of lncRNAs extends to the modulation of the tumor microenvironment, thereby affecting drug resistance. One such lncRNA is LUCAT1 (Lung Cancer Associated Transcript 1, chromosome 5q14.3), originally identified as Smoke and Cancer Associated lncRNA-1 (SCAL1), which has been shown to be involved in the post-transcriptional regulation of multiple key oncogenes [45, 46]. Recently, accumulating research indicates that LUCAT1 plays a favorable role in post-transcriptional regulation of multiple key oncogenes [47], and dysregulated expression of LUCAT1 has been connected to cancer stem cells (CSCs) [48, 49]. Despite this, the underlying mechanism of LUCAT1 contributing to cancer the occurrence and progression, particularly in chemoresistance of BC, remains elusive.

Cancer organoid model is an effective preclinical cancer model constructed from tumor tissues, which could faithfully represent clinical manifestations and validate the efficacy of candidate therapeutic regimens [50–52]. Given their ability to accurately mimic the in vivo tumor microenvironment, organoid-based models serve as an indispensable tool for identifying key molecular mechanisms of chemoresistance and developing targeted strategies to overcome therapy resistance in bladder cancer. In the present study, cancer organoid models were developed from urothelial carcinomas to explore the pathophysiology mechanism of chemoresistance of UC and validate the efficacy of candidate therapeutic regimens, and RNA-seq was performed to screen for lncRNAs involved in chemoresistance of BC. Moreover, to investigate the underlying mechanism of chemoresistance in BC, GEM-resistant (GR) cell lines were established by in vivo GEM chemotherapy induction simulating the biological processes of chemoresistance. Therefore, we performed an unbiased analysis of the GR-organoids and found that LUCAT1 expression was significantly up-regulated in BC. Furthermore, LUCAT1 was also highly expressed in GR-BC cells and urinary exosomes of patients after chemotherapy. Moreover, GR-BC cells enhanced the stemness phenotype and chemoresistance of BC cells by delivering LUCAT1 through exosome. Mechanistically, LUCAT1 could significantly up-regulate HMGA1 expression, a key regulatory genes of cancer stem cell, and inhibition of malignant phenotypes in BC cells by silencing LUCAT1 was reversed by overexpression of HMGA1. Moreover, further experimental results demonstrated that LUCAT1 could bind to IGF2BP2 (the mRNA binding protein of HMGA1) and up-regulated stability of HMGA1 mRNA in an m6A-dependent manner. Collectively, our findings highlight that LUCAT1 an effective tumour biomarker, with promising clinical implications as a therapeutic and diagnostic target of BC.

## Materials and methods

### Clinical sample collection, cell and organoid culture

Fresh bladder cancer tissue samples and pair-matched normal tissue samples were obtained from patients who underwent radical cystectomy. Following previously reported protocols, clinical samples were collected, and organoids were cultivated [53, 54]. Supplementary Table 1 highlights the characteristics of the patients. Cells were cultured according to previously standard protocols. The normal urothelial cell line and bladder cancer (BC) cell lines used in this study were obtained from the Institute of Cell Research, Chinese Academy of Sciences, located in Shanghai, China.

### Cell transfection, RNA extraction and quantitative real-time PCR

The plasmid vectors used in this investigation were acquired from BioVector NTCC, Inc., Guangzhou, China. The establishment of the stable cell line was carried out by lentivirus infection following established protocols. Cell transfection, RNA extraction, and qRT-PCR were conducted according to previously standard protocols. Supplementary Table 2 provides details on the primer sequences used in this investigation.

### Cell self-renewal assays

The cell self-renewal ability was assessed using established methodologies. Specifically, the tumour sphere formation assays were utilized to evaluate the ability of BC cells to proliferate and form tumour spheres, while the single-cell tumour sphere formation assays were employed to assess the ability of these cells to form three-dimensional spheroid structures. These assays were performed in accordance with previously standard protocols.

### Chemosensitivity assay

Flow cytometry analysis and TdT-mediated dUTP Nick-End Labeling (TUNEL) assay were utilized to assess the chemosensitivity of BC cells through determining cell apoptosis induced by GEM. Briefly, the corresponding cells were cultured in normal medium with GEM or PBS, and collected after incubation for 48 h. Cell apoptosis was detected using was determined by PE Annexin V apoptosis detection kits (BD Pharmingen, San Diego, CA, USA) in accordance with the manufacturer’s instructions. Finally, cell apoptosis was determined using flow cytometry (EPICS, XL-4, Beckman, CA, USA) according to standard protocols. Cell apoptosis induced by GEM was also determined using a TUNEL in vitro kit (RiboBio, Guangzhou, China) in accordance with the manufacturer’s instructions. Finally, the fluorescence of cells was observed using fluorescence microscopy.

### Mouse model experiments

All animal experiments adhered to ethical guidelines and recommendations set forth by the Institutional Animal Care and Use Committee (IACUC) of The First Affiliated Hospital of Zhengzhou University, which provided approval. Male BALB/c nude mice aged five weeks were sourced from Vital River (Beijing, China) and housed in a facility with a specific pathogen-free (SPF) barrier. Supplementary Materials and Methods section contains detailed methods and procedures employed in both the tumor-initiating capacity assay and in vivo chemotherapy assay.

### Statistical analyses

Statistical significance was determined using Student’s t-test or chi-squared test for quantitative data from three independent experiments, with results expressed as the mean ± standard deviation. To evaluate cumulative survival probability, Kaplan-Meier survival analysis was employed, and a *p* value of < 0.05 were considered statistically significant. All statistical tests were performed using SPSS 19.0 software (SPSS Inc. Chicago, IL, USA).

## Results

### Establishment of UC organoids to screen LncRNAs involved in chemoresistance of BC

**Fig. 1:**
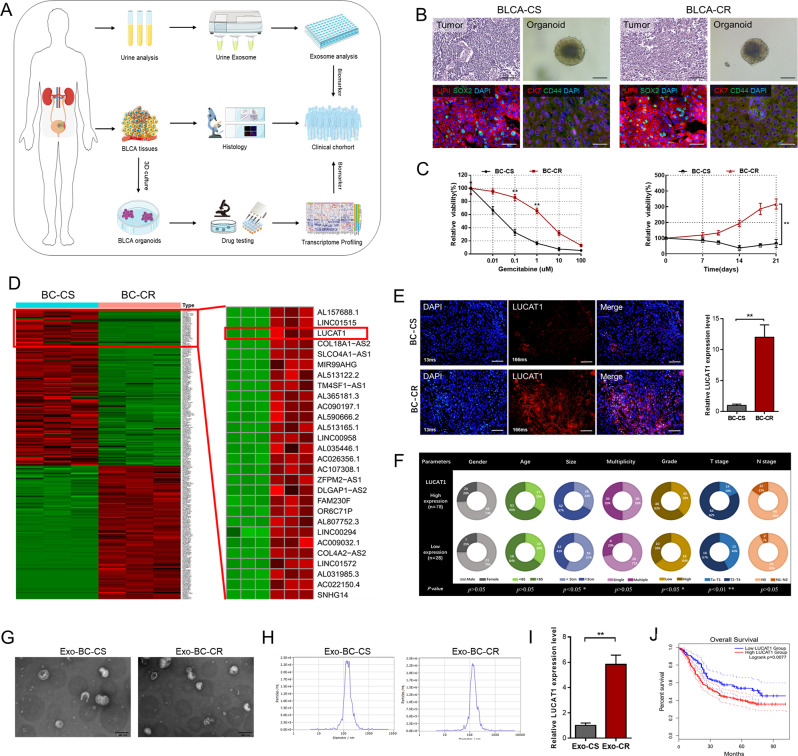
Establishment of UC organoids to screen lncRNAs involved in chemoresistance of BC. **A**: Establishment of patient-derived UC organoids to explore the pathophysiology mechanism of chemoresistance and validate the efficacy of candidate therapeutic regimens. **B**: Representative H&E staining images, immunofluorescence images (CK7, UPII, SOX2, CD44) and bright-field images of organoids. **C**: Dose-response curves of UC organoids treated with GEM in vitro and in vivo. **D**: The lncRNA candidates in expression profile analyses were showed in the thermograph included LUCAT1. E: FISH analyses showed that LUCAT1 expression is highly expressed in the GEM-resisitant xenografts. F: LUCAT1 expression was correlated with clinical characteristics of BC. G: Urinary exosomes of BC patients were identified using Nanoparticle Tracking Analysis (NTA). H: Urinary exosomes of BC patients were identified using electronic speculum. I: LUCAT1 is significantly upregulated in urinary exosomes of BC patients after chemotherapy. J: Higher LUCAT1 expression is related to BC patients’ shorter OS in TCGA-BLCA dataset (http://gepia2.cancer-pku.cn/#index). The data are shown as the mean ± SD. *p < 0.05; **p < 0.01

For the evaluation of candidate therapeutic regimens, cancer organoids have been developed from a variety of tumors, including urothelial carcinomas [53]. To explore the pathophysiology mechanism of chemoresistance of BC, we collected clinical samples to establish patient-derived UC organoids (Fig. 1A) and performed hematoxylin-eosin (H&E) and immunofluorescence (IF) analyses on organoid pairs to assess the expression levels of urothelial carcinoma and the stemness markers (Fig. 1B). In Fig. 1C, there are dose-response curves of UC organoids treated with GEM in vitro and in vivo. We conducted comprehensive lncRNAs expression profile analyses to identify lncRNAs involved in chemoresistance of bladder cancer and found LUCAT1 expression is significantly up-regulated in chemotherapy-resistant BC (BC-CR) cells compared to chemotherapy-sensitive BC (BC-CS) cells (Fig. 1D). Consistently, FISH analyses showed that LUCAT1 expression was markedly up-regulated in the BC-CR xenografts (Fig. 1E). To investigate the clinical relevance of LUCAT1 in BC, we determined the expression level of LUCAT1 in a total of 106 patients with urothelial carcinoma. Remarkably, LUCAT1 was significantly up-regulated in bladder cancer tissues comparing to paired nonneoplastic tissues (Fig. S1A), and a positive correlation was observed between higher LUCAT1 expression and advanced T stage as well as higher histological grade (Fig. 1F and S1B) and poor prognosis (Fig. 1J). Furthermore, we collected and identified urinary exosomes of BC patients to explored the clinical relevance of LUCAT1 in the efficacy of chemotherapy (Fig. 1G and H), and found LUCAT1 was significantly upregulated in urinary exosomes of BC patients after chemotherapy (Fig. 1I). As shown in Supplementary Table 1, LUCAT1 expression is associated with clinical pathological characteristics of bladder urothelial carcinoma patients. In summary, we identified that LUCAT1 was up-regulated in the BC-CR organoids and LUCAT1 has the potential to serve as a prognostic marker for BC patients.

### Gemcitabine chemotherapy enriches stem-like cells in BC

**Fig. 2:**
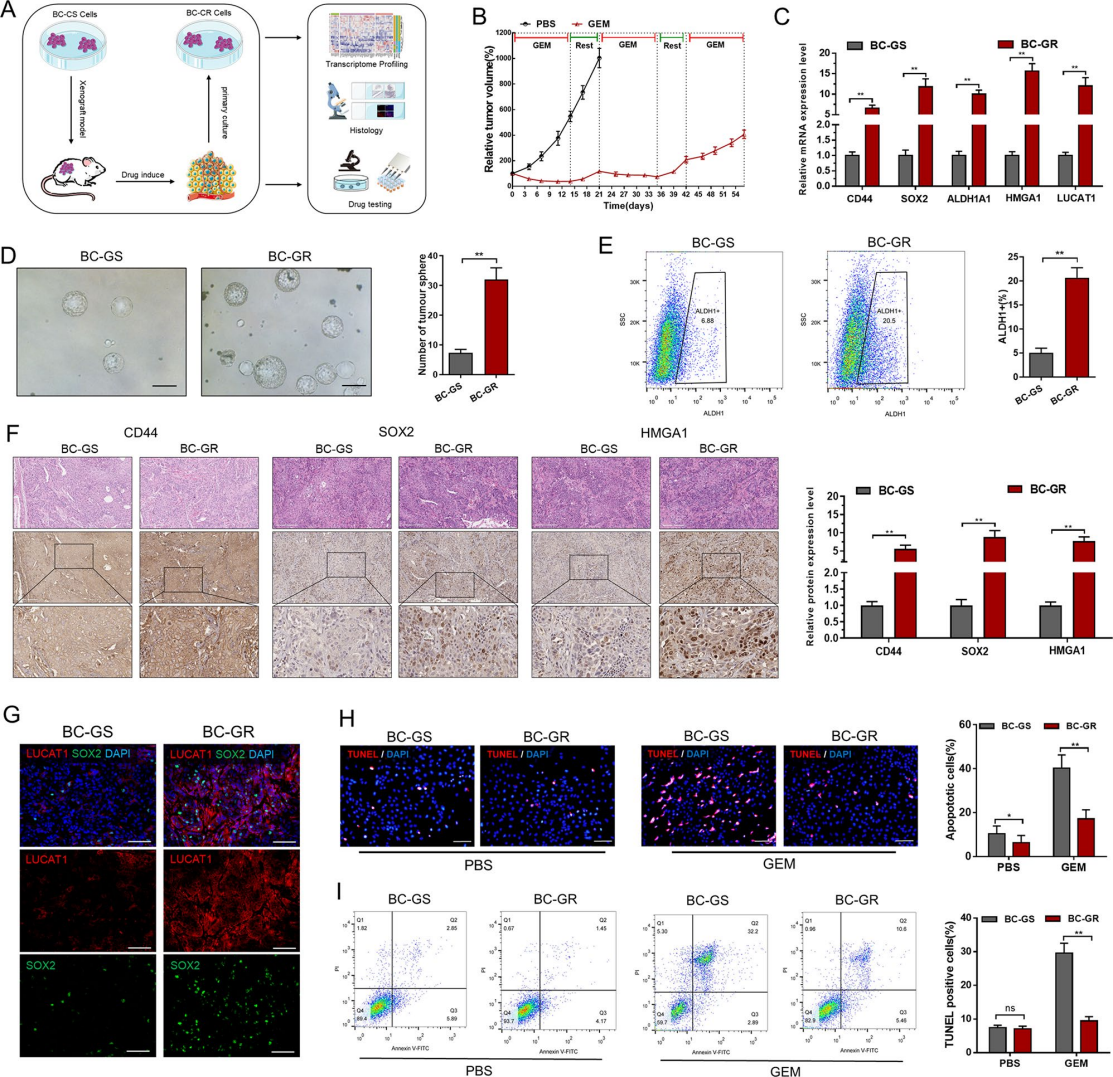
Gemcitabine chemotherapy enriches stem-like cells in bladder cancer. **A** and **B**: GEM-resisitant BC cell models were established via mimicking the clinical regimen with multiple treatment cycles of GEM (40 mg/kg) and gap periods. **C**: RT-qPCR analyses demonstrated high expression of stemness markers and LUCAT1 in the GEM-resisitant (GR) xenografts compared to GEM-sensitive (GS) xenografts. **D**: The stemness of BC cells isolated from xenografts were evaluated using tumour sphere formation assay. **E**: The isolation of BCSCs from BC cells was carried out through flow cytometry, utilizing the stem cell markers ALDH1. **F**: Highly expressed stemness markers were detected in the GEM-resistant xenografts through IHC analyses. **G**: FISH and immunofluorescence analyses showed that LUCAT1 and stemness markers were elevated in the GEM-resistant xenografts. **H** and **I**: TUNEL assay (**H**) and flow cytometry analyses (**I**) showed that chemosensitivity of GR-BC cells were decreased compared to the GS-BC cells group. The results are shown as the mean ± SD. *p < 0.05; **p < 0.01

To mimic the biological process of chemotherapy resistance in bladder cancer (Fig. 2A), GEM-resistant BC cell model was established by recreating clinical regimens comprising of multiple GEM treatment cycles and gap periods (Fig. 2B). Then cancer cells were separated from xenografts and the efficiency of tumour sphere formation of GEM-resistant BC (BC-GR) cells was increased compared to GEM-sensitive BC (BC-GS) cells (Fig. 2D). Utilizing flow cytometry, bladder cancer stem cells (BCSCs) were separated from BC cells using the stemness marker ALDH1, and the proportion of BCSCs in GR-BC cells was higher compared to the vehicle group (Fig. 2E). Consistently, RT-qPCR and IHC analyses demonstrated that the stemness markers CD44 and SOX2 were expressed at elevated levels in the GEM-resistant xenografts (Fig. 2C and F), and FISH and immunofluorescence analyses also showed that elevated LUCAT1 expression and stemness markers were detected in the GEM-resistant xenografts (Fig. 2G). Furthermore, TUNEL assay and flow cytometry analyses showed that chemosensitivity of GEM-resistant BC cells were decreased compared to vehicle group (Fig. 2H and I). Collectively, these data suggest that GEM therapy may increase the number of CSCs in BC.

### LUCAT1 promotes the stemness phenotype and chemoresistance of BC cells

**Fig. 3:**
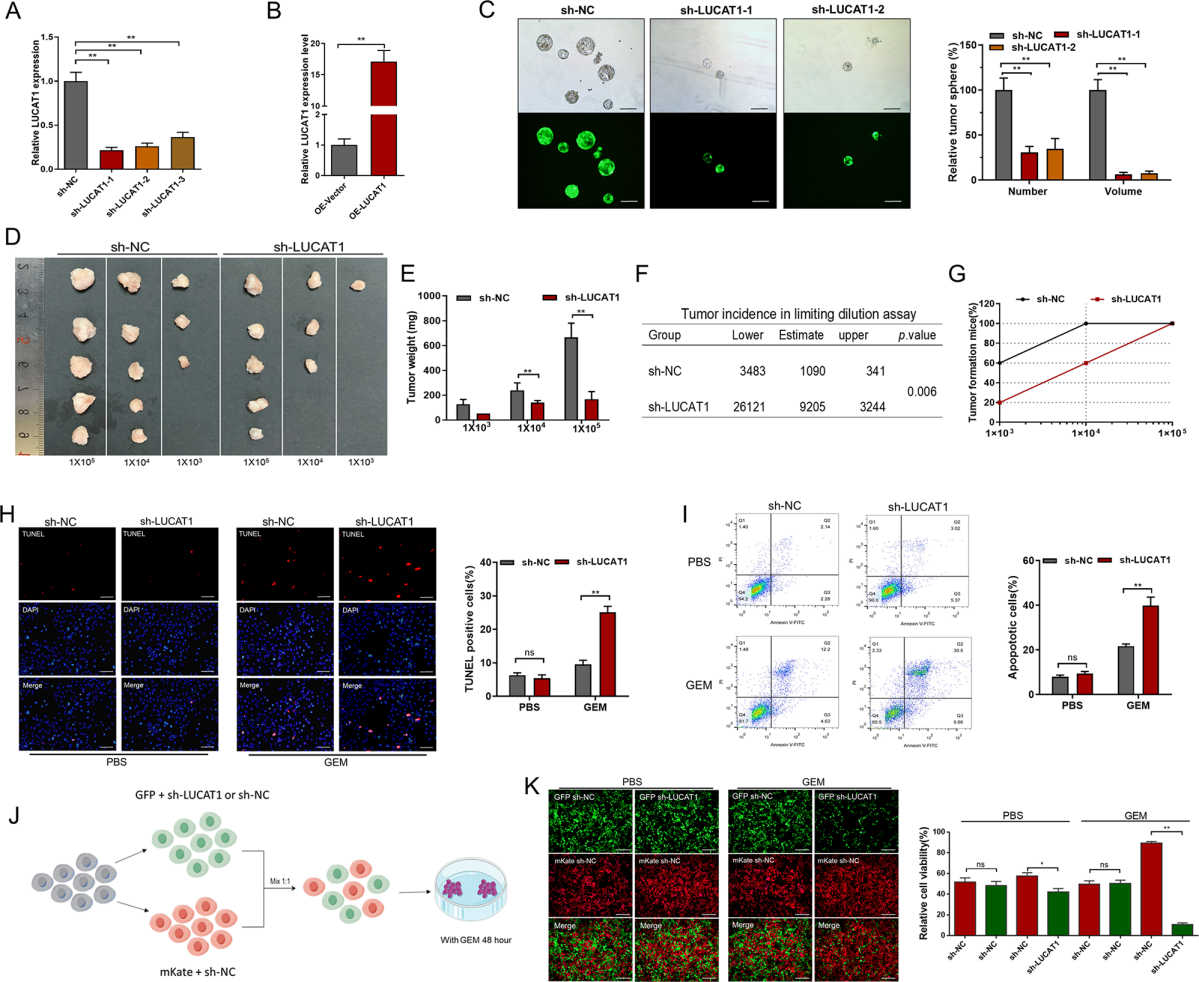
LUCAT1 promotes the stemness phenotype and chemoresistance of BC cells. **A**: LUCAT1-specific shRNA downregulated LUCAT1 expression in BC cells. **B**: LUCAT1 expression vector upregulated LUCAT1 expression in BC cells. **C**: The spheroid-formation ability of BC cells were determined using tumour sphere formation assay. **D**: The tumourigenicity capacity of BC cells were determined using in vivo tumour initiation assay. **D** and **E**: Knockdown of LUCAT1 using sh-LUCAT1 (sh-LUCAT1-1) decreased the size and weight of xenografts. **F** and **G**: The extreme limiting dilution analysis (ELDA) was performed to determine tumourigenicity capacity of BC cells. Knockdown of LUCAT1 decreased the incidence of xenografts. **H**: TUNEL assay was conducted to investigate the impact of GEM on cell apoptosis. **I**: Flow cytometry analysis was conducted to investigate the effects of GEM on cell apoptosis. **J**: The cell fluorescence trace system was utilized to measure the chemosensitivity of BC cells in diverse treatment groups. **K**: The proportion of LUCAT1 down-regulated cells was decreased in GEM-treatment group. The data are shown as the mean ± SD. *p < 0.05; **p < 0.01

To determine whether LUCAT1 is involved in regulation of stemness phenotype in BC cells, we used LUCAT1-specific shRNAs (Fig. 3A) and LUCAT1-specific expression vector (Fig. 3B) to up- and down-regulate LUCAT1 expression in BC cells, respectively. A tumour sphere formation assay was performed to determine the spheroid-formation ability of BC cells. Knockdown (Fig. 3C) and overexpression (Fig. S1C) of LUCAT1 decreased and increased the number and size of tumor spheres, respectively. Furthermore, a tumour initiation assay was performed to further evaluate the tumorigenicity capacity of BC cells in vivo, and knockdown of LUCAT1 in BC cells decreased the size (Fig. 3D), weight (Fig. 3E) and incidence (Fig. 3F and G) of xenografts. Moreover, IHC analyses showed that knockdown of LUCAT1 decreased the expression of stemness marker CD44 and the proliferation marker Ki67 in BC cells in the xenografts (Fig. S1D). Due to previous evidence showing that a stemness phenotype can induce GEM chemoresistance and that LUCAT1 can promote the stemness of BC cells, further experiments were performed to determine whether LUCAT1 regulates the chemoresistance of BC cells. The effect of GEM treatment on cell apoptosis was evaluated via TUNEL assay and flow cytometry analysis. Moreover, knockdown (Fig. 3H and I) and overexpression (Fig. S1E and S1F) of LUCAT1 increased and decreased the proportion of apoptotic cells, respectively. Furthermore, a cell fluorescence tracing system was utilized to evaluate the chemosensitivity of BC cells in various treatment groups (Fig. 3J). As shown in Fig. 3K, the proportion of LUCAT1-downregulated cells was decreased in the GEM-treated group in vitro. Taken together, these findings indicate that LUCAT1 promotes chemoresistance in bladder cancer cells by enhancing their stemness.

### Exosome-transmitted LUCAT1 promotes the stemness and chemoresistance of BC cells

**Fig. 4:**
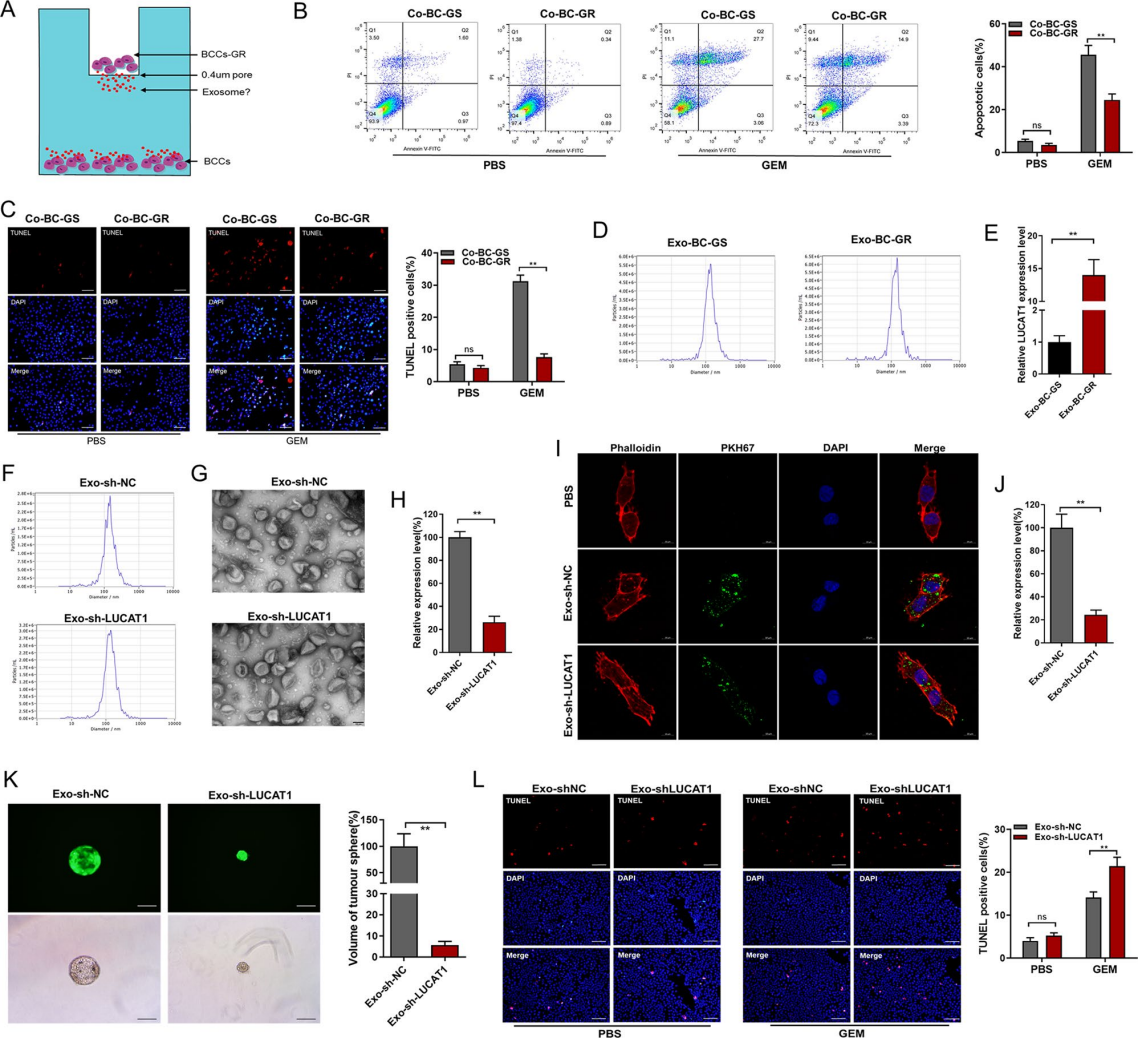
Exosome-transmitted LUCAT1 promotes stemness and chemoresistance of BC cells. **A**: Schematic diagram of co-culture with GEM-resistant BC cells. **B**: Flow cytometry analysis was conducted to assess the impact of GEM on cell apoptosis. **C**: Cell apoptosis induced by GEM were evaluated using TUNEL assay in various treatment groups. **D**: Exosomes isolated from the culture medium of BC-GS and BC-GR cells were identified using Nanoparticle Tracking Analysis. **E**: LUCAT1 is significantly up-regulated in exosomes isolated from the culture medium of GEM-resistant BC cells. F and G: Exosomes separated from BC cell culture medium were identified using Nanoparticle Tracking Analysis (**F**) and electron microscope (**G**). **H**: LUCAT1 is significantly down-regulated in exosomes separated from the culture medium of LUCAT1 knockdown group compared to control group. **I**: The confocal laser-scanning microscope was employed to observe internalization of exosomes labeled with PKH67. **J**: The successful uptake of exosomes derived from LUCAT1 knockdown group did not increased LUCAT1 expression in BC cells. **K**: The changes in stemnesss of BC cells were assessed using single-cell tumour sphere formation assays. **L**: The changes in GEM chemosensitivity of BC cells in different exosome uptake groups were determined using TUNEL assay. The results are shown as the mean ± SD. *p < 0.05; **p < 0.01

Remarkably, we found that cocultures consisting of GEM-resistant BC cells and non-resistant BC cells (Fig. 4A) enhanced the chemoresistance of the BC cells (Fig. 4B and C). As exosomes are essential messengers in cell-to-cell communication and recent studies suggested that exosomes originating from CSCs facilitate drug resistance by transferring miRNAs [32], we hypothesized that exosomes derived from BCSCs may transfer LUCAT1 and thus facilitate the horizontal transfer of drug-resistant factors. Moreover, exosomes were isolated from the medium of BC-GS and BC-GR cell cultures (Fig. 4D), and LUCAT1 was significantly upregulated in the presence of exosomes from GEM-resistant BC cells (Fig. 4E). To investigate whether exosome-transmitted LUCAT1 mediates GEM chemoresistance in BC cells, we isolated exosomes from the medium of BC cell cultures, identified the exosomes via nanoparticle tracking analysis (NTA) (Fig. 4F and S2B) and electron microscopy (Fig. 4G and S2C). We found that LUCAT1 was decreased in exosomes derived from the LUCAT1 knockdown group (Fig. 4H) and enriched in exosomes derived from the LUCAT1 overexpression group (Fig. S2D). We observed that exosomes fluorescently labelled with PKH67 were endocytosed by BC cells (Fig. 4I and S2E). The successful uptake of exosomes derived from the LUCAT1 overexpression group increased LUCAT1 expression in BC cells (Fig. S2F), while the successful uptake of exosomes derived from the LUCAT knockdown group did not lead to an increase in LUCAT1 expression in BC cells (Fig. 4J). We further determined the changes in the stemness phenotypes of BC cells via single-cell tumour sphere formation assays and found that the uptake of LUCAT1-decreased exosomes did not enhanced spheroid formation (Fig. 4K) by BC cells. Conversely, the uptake of LUCAT1-enriched exosomes enhanced spheroid formation (Fig. S2H) by BC cells. Furthermore, changes in GEM chemosensitivity of BC cells in different exosome uptake groups were determined by TUNEL assay and flow cytometry analysis. The results depicted in Fig. 4L and S2A indicated that the uptake of LUCAT1-depleted exosomes failed to reduce cell apoptosis induced by GEM compared to that in the vehicle group. Moreover, the uptake of LUCAT1-enriched exosomes decreased cell apoptosis induced by GEM in BC cells (Fig. S2G and S2I). These findings indicate that exosomes derived from stem-like (chemotherapy-resistant) BC cells enhance the stemness and chemoresistance of BC cells by delivering LUCAT1.

### LUCAT1 promotes the stemness phenotype of BC cells by modulating HMGA1

**Fig. 5:**
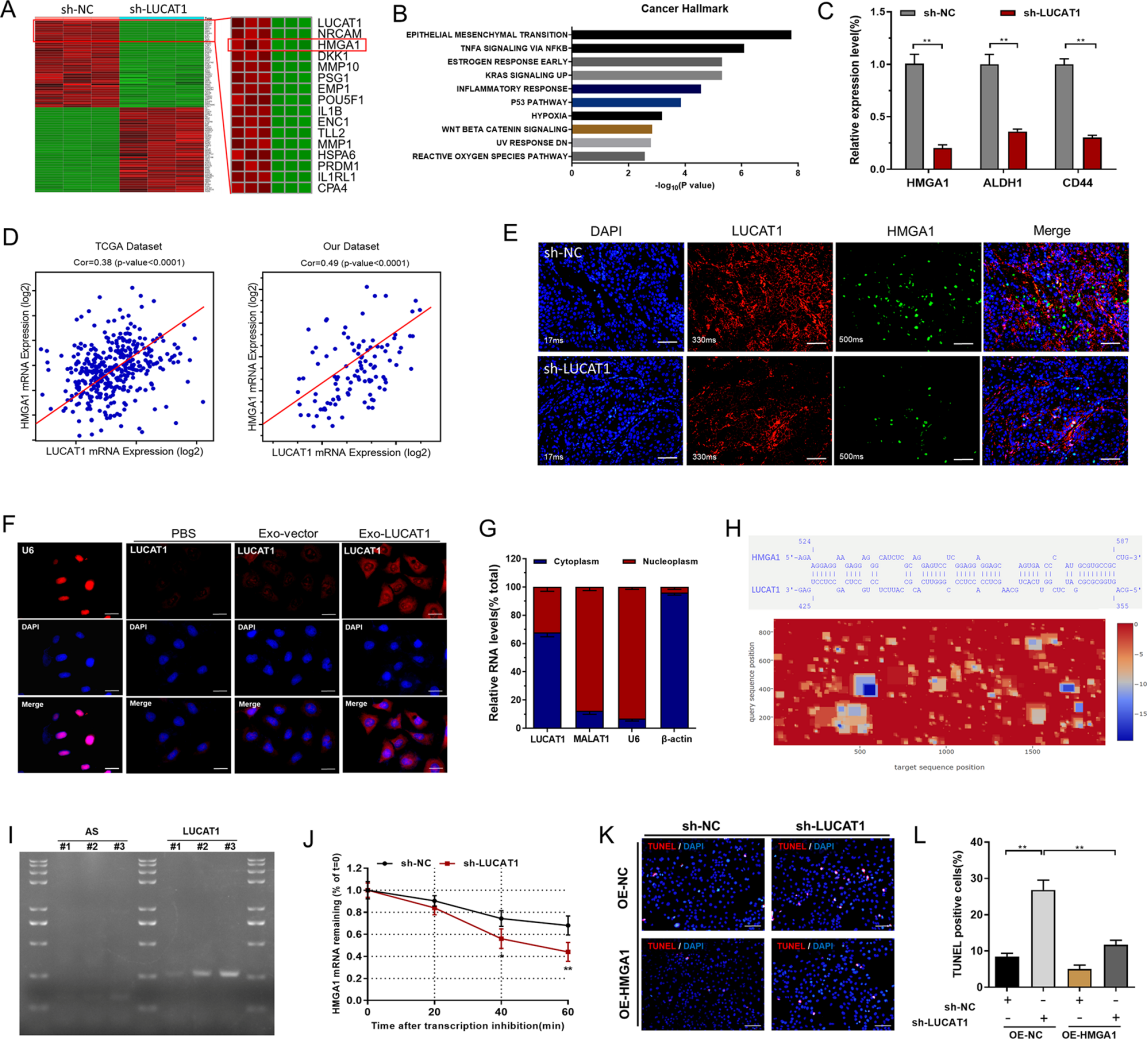
LUCAT1 promotes the stemness phenotype of BC cells by modulating HMGA1. **A**: The differentially expressed candidates in expression profile analyses were displayed in the thermograph. **B**: LUCAT1-upregulated genes were enriched in key cancer signaling pathways analyzed by cancer hallmark. **C**: Knockdown of LUCAT1 decreased HMGA1 and CSC markers expression in BC cells. **D**: LUCAT1 expression positively correlated with HMGA1 in BC. **E**: FISH and immunofluorescence analyses were used to determine LUCAT1 and HMGA1 expression in xenografts. F and G: RNA FISH (**F**) and qRT-PCR (**G**) were employed to assess the subcellular localization of lncRNAs. H: Bioinformatics analysis revealed the presence of common putative binding sites between LUCAT1 and HMGA1 mRNA, and minimal energy of LUCAT1-HMGA1 was showed in the thermograph. I: RNA pulldown and PCR showed the interaction between LUCAT1 and HMGA1 mRNA. J: Knockdown of LUCAT1 increased HMGA1 mRNA degradation in BC cells treated with actinomycin D (5 µg/ml). K and L: The inhibition of chemoresistance induced by silencing LUCAT1 was reversed by overexpression of HMGA1. The results are shown as the mean ± SD. *p < 0.05; **p < 0.01

To explore the potential mechanisms of LUCAT1-mediated biological processes, extensive transcriptional analysis was conducted using TCGA and CCLE datasets. Our analysis showed that LUCAT1 expression was positively associated with CSC markers in BC (Fig. S3A), and the LUCAT1-correlated genes enriched in key tumor signaling pathways (Fig. S2B-E). To identify potential downstream target genes regulated by LUCAT1, we performed RNA-seq analysis (Fig. 5A). The results demonstrated that knockdown of LUCAT1 led to the downregulation of multiple key tumor regulatory genes, including HMGA1 (Fig. 5A and C). Furthermore, pathway enrichment analysis revealed that these downregulated genes were significantly enriched in essential cancer-related signaling pathways (Fig. 5B). These assays revealed a positive correlation between LUCAT1 and HMGA1 in BC, and this correlation was verified in clinical BC tumour samples (Fig. 5D). FISH and immunofluorescence analyses showed that LUCAT1 and HMGA1 expression was significantly elevated in xenografts treated with GEM, and co-expression of LUCAT1 and HMGA1 was detected in GEM-resistant BC cells (Fig. 5E and S4A). Accordingly, the biological function and potential molecular roles of lncRNAs are closely linked to their subcellular localization. Both RNA-FISH (Fig. 5F) and qRT-PCR (Fig. 5G) analyses demonstrated that LUCAT1 was predominantly located in cytoplasm of BC cells, which suggested that LUCAT1 is a candidate post-transcriptional regulator of gene expression. Bioinformatics analyses revealed common putative binding sites in LUCAT1 and HMGA1 mRNA, and the minimal energy of LUCAT1-HMGA1 mRNA binding was showed in the thermograph (Fig. 5H). The interaction between LUCAT1 and HMGA1 mRNA was verified by further experimental results (Fig. 5I and S4B). Notably, LUCAT1 may decrease HMGA1 mRNA degradation in BC cells (Fig. 5J and S4C). We conducted further research to determine whether LUCAT1 modulates the stemness of BC cells via an HMGA1-dependent mechanism. The results of our study indicated that the inhibition chemoresistance and stemness (Fig. 5K-L and S4D-G) induced by silencing LUCAT1 in BC cells were significantly reversed by HMGA1 overexpression. Our findings suggest that LUCAT1 enhances the stemness of BC cells through an HMGA1-dependent mechanism.

### LUCAT1 positively regulates HMGA1 expression by interacting with IGF2BP2

**Fig. 6:**
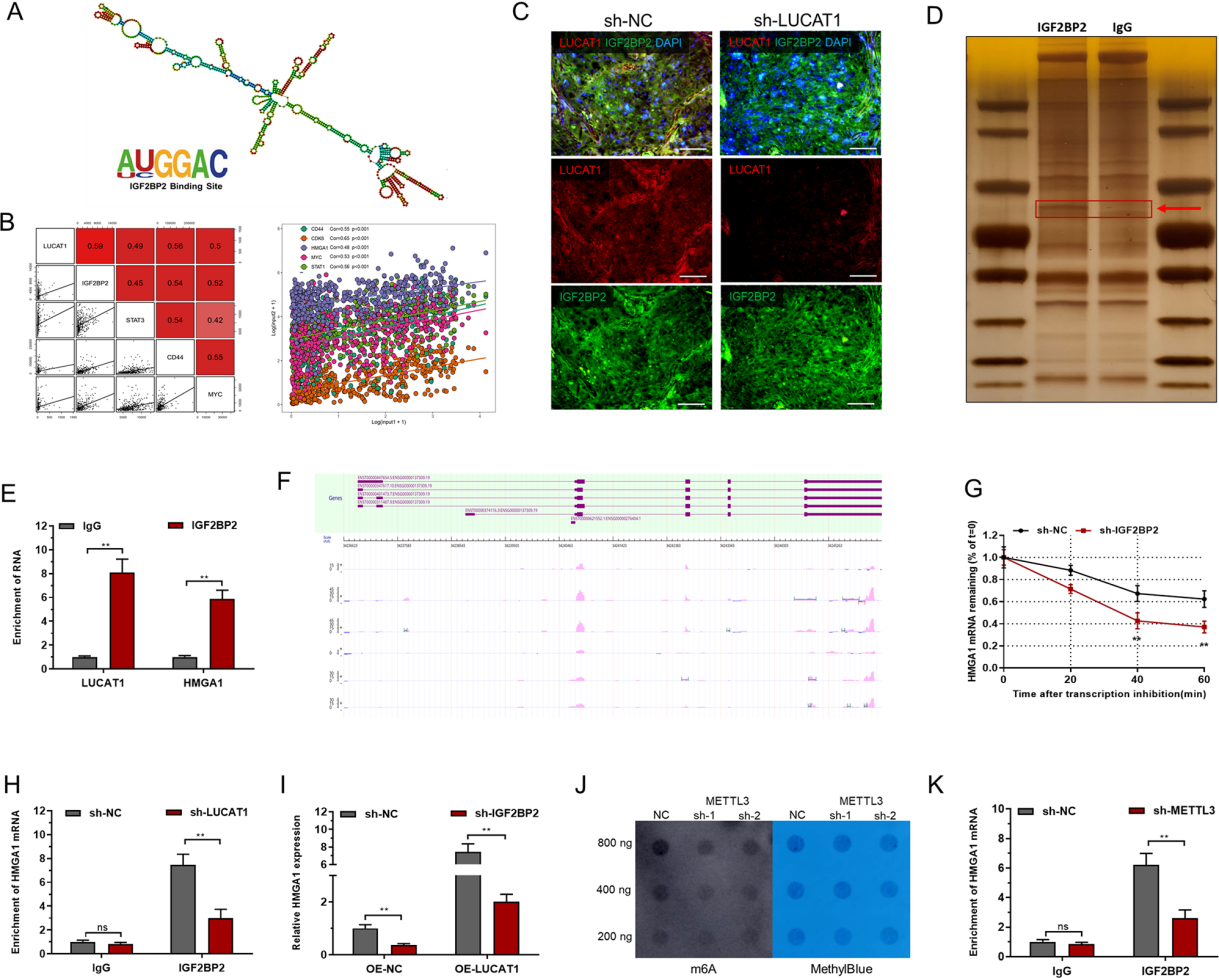
LUCAT1 positively regulates HMGA1 expression by interacting with IGF2BP2. **A**: Sequence analysis of LUCAT1 by POSTAR2 identified a sequence motif and structural preference in the RNA binding protein (RBP) binding site for IGF2BP2. **B**: IGF2BP2 expression positively correlated with LUCAT1, HMGA1, CD44 and MYC expression in BC dataset. **C**: FISH and immunofluorescence analyses showed that LUCAT1 and IGF2BP2 were colocalized in BC cells, but knockdown of LUCAT1 unreduced the expression level of IGF2BP2 in vivo. **D**: RNA immunoprecipitation (RIP) was performed using IGF2BP2 and showed an obvious 60–70 kDa band. **E**: RNA immunoprecipitation (RIP) showed enrichment of LUCAT1 and HMGA1 by IGF2BP2. **F**: RIP-seq showed the interaction between IGF2BP2 and HMGA1 mRNA. G: Knockdown of IGF2BP2 increased HMGA1 mRNA degradation in BC cells treated with actinomycin D (5 µg/ml). H: Knockdown of LUCAT1 decreased the enrichment of HMGA1 by IGF2BP2. I: Knockdown of IGF2BP2 decreased the up-regulation of LUCAT1 on HMGA1 in BC cells. J: Knockdown of METTL3 decreased m6A levels of total mRNA in BC cells. K: Knockdown of METTL3 decreased the enrichment of HMGA1 mRNA by IGF2BP2. The results are shown as the mean ± SD. *p < 0.05; **p < 0.01

To explore the regulatory mechanisms underlying the effect of LUCAT1 on HMGA1, RNA pull-down assays were utilized to investigate RNA binding proteins of LUCAT1. In Fig. 6A, the sequence analysis of LUCAT1 using POSTAR2 revealed a sequence motif and structural preference in the RNA binding protein (RBP) binding site for IGF2BP2 (Fig. 6A). In vitro RNA pull-down assays with biotinylated LUCAT1 and antisense control showed an obvious 60–70 kDa band (Fig. S5A), and subsequent experiments confirmed that LUCAT1 interacts with the RNA-binding protein IGF2BP2. Furthermore, IGF2BP2 expression positively correlated with LUCAT1, HMGA1 and cancer stem cell markers expression in BC cells (Fig. 6B and S5B). FISH and immunofluorescence analyses showed that LUCAT1 and IGF2BP2 were colocalized in BC cells, but knockdown of LUCAT1 unreduced the expression level of IGF2BP2 in vivo (Fig. 6C). RNA immunoprecipitation (RIP) assays using IGF2BP2 revealed an obvious 60–70 kDa band (Fig. 6D) and demonstrated significant enrichment of LUCAT1 and HMGA1 mRNA (Fig. 6E), confirming the interaction between LUCAT1 and IGF2BP2. Furthermore, RIP-seq analysis confirmed the interaction between IGF2BP2 and HMGA1 mRNA (Fig. 6F), and knockdown of IGF2BP2 increased HMGA1 mRNA degradation in BC cells treated with actinomycin D (Fig. 6G and S5C). Subsequent experiments confirmed that knockdown of LUCAT1 led to a decrease in the enrichment of HMGA1 mRNA by IGF2BP2 (Fig. 6H). Moreover, knockdown of IGF2BP2 decreased the up-regulation of LUCAT1 on HMGA1 in BC cells (Fig. 6I). As IGF2BP2 was reported to functions as a m6A reader, we measured m6A levels of HMGA1 mRNA by MeRIP-qPCR in BC cells to further explore the link between IGF2BP2 and HMGA1 (Fig. S5D). Methyltransferase-like 3 (METTL3) is a key RNA N6-adenosine methyltransferase in BC, and we found knockdown of METTL3 decreased m6A levels of total mRNA in BC cells (Fig. 6J). Further experiments found knockdown of METTL3 decreased the enrichment of HMGA1 mRNA by IGF2BP2 (Fig. 6K), and decreased the up-regulation of IGF2BP2 on HMGA1 (Fig. S5E). The findings suggest that LUCAT1 interacts with IGF2BP2 in a m6A-dependent manner to positively regulate HMGA1 expression.

### Exosome-transmitted LUCAT1 promotes GEM chemoresistance of BC cells in vivo

**Fig. 7:**
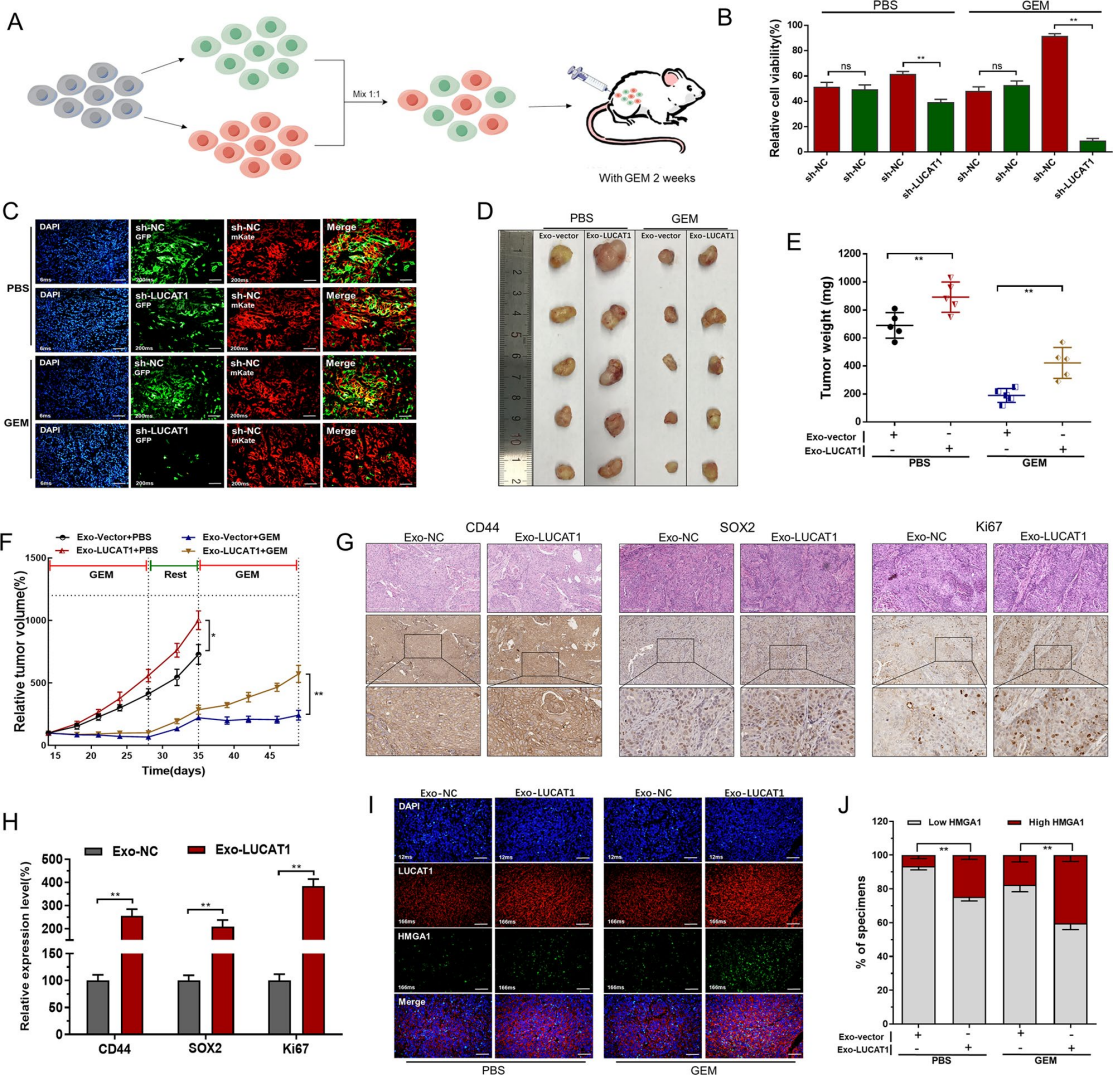
Exosome-transmitted LUCAT1 promotes GEM chemoresistance of BC cells in vivo. **A**: Schematic diagram of cell fluorescence trace system in vivo. The chemosensitivity of BC cells in different treatment groups was also determined using a cell fluorescence trace system in vivo. **B** and **C**: The proportion of LUCAT1 down-regulated cells was decreased in tumor xenografts of GEM-treatment group. **D**: The effects of exosome-transmitted LUCAT1 on chemosensitivity of BC cells was determined by establishing xenografts in BALB/c nude mice models. Xenografts collected from mice were exhibited and measured. **E**: The tumour weight of LUCAT1-enriched exosomes treatment group was greater than that of control groups. **F**: Tumour growth of LUCAT1-enriched exosomes treatment group was faster than that of control groups, and chemoresistance in LUCAT1-enriched exosomes treatment group was enhanced compared with control groups. G and H: Immunohistochemistry analyses showed that exosome-transmitted LUCAT1 upregulated stemness markers and proliferation marker Ki67 expression in BC cells. **I** and **J**: FISH and immunofluorescence analyses showed that LUCAT1 and HMGA1 were co-expressed in BC cells in vivo. The data are shown as the mean ± SD. *p < 0.05; **p < 0.01

**Fig. 8:**
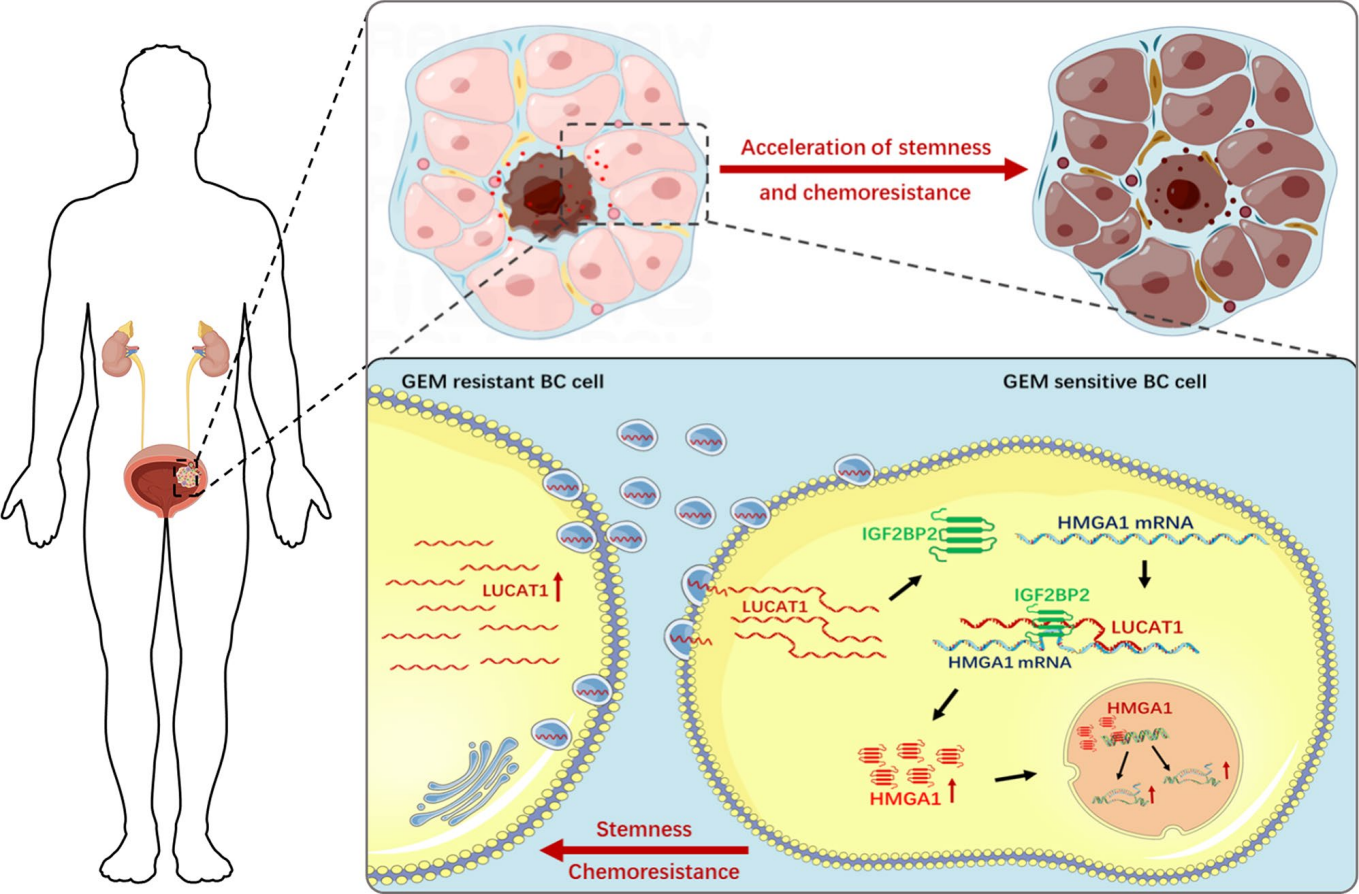
Schematic diagram of the oncogenic role of exosome-transmitted LUCAT1 in bladder cancer. Exosome-transmitted LUCAT1 promotes the stemness phenotype and chemoresistance of BC cells via upregulating HMGA1 expression via binding to IGF2BP2, thus contributing to its oncogenic activity in bladder cancer pathogenesis

To explore potential biological functions and mechanisms of LUCAT1-mediated in vivo, we established a mouse chemotherapy model. A cell fluorescence trace system was utilized to evaluate the chemosensitivity of BC cells in different treatment groups in vivo (Fig. 7A). We found the proportion of LUCAT1 down-regulated cells was decreased in tumor xenografts of GEM-treatment group (Fig. 7B and C). Furthermore, we determined the effects of exosome-transmitted LUCAT1 on chemosensitivity of BC cells by establishing xenografts in BALB/c nude mice models. As shown in Fig. 7D, xenografts collected from mice were exhibited and measured (Fig. 7D). The tumour weight of LUCAT1-enriched exosomes treatment group was greater than which of control groups (Fig. 7E). Tumour growth in LUCAT1-enriched exosomes treatment group was faster than that of control groups, and chemoresistance in LUCAT1-enriched exosomes treatment group was enhanced compared with control groups (Fig. 7F). We found that exosome-transmitted LUCAT1 upregulated stemness markers and proliferation marker in BC cells (Fig. 7G and H). Moreover, FISH and immunofluorescence analyses showed that LUCAT1 and HMGA1 were co-expressed in BC cells (Fig. 7I and J) in vivo. The results indicated that LUCAT1 promotes GEM chemoresistance of BC cells in vivo. As shown in Fig. 8, exosome-transmitted LUCAT1 promotes the stemness phenotype and chemoresistance of BC cells via upregulating HMGA1 expression via binding to IGF2BP2, thus contributing to its oncogenic activity in bladder cancer pathogenesis.

## Discussion

The development of chemotherapy resistance poses a major therapeutic challenge in the management of bladder cancer, necessitating the exploration of novel therapeutic strategies to overcome this barrier [55]. Research on chemotherapy resistance in bladder cancer is an active area of investigation. Chemotherapy resistance in bladder cancer is an area of intensive research, with multiple mechanisms proposed to explain its development, including alterations in drug transporters, DNA repair mechanisms, and signaling pathways involved in cell survival and proliferation [56–58]. However, despite these insights, the precise molecular and cellular processes governing chemoresistance and tumor relapse remain incompletely understood. There is growing evidence supports the notion that cancer stem cells (CSCs), which represent a minor fraction of the heterogeneous tumor mass, play a significant role in tumor recurrence and chemotherapy resistance [58, 59]. More importantly, chemotherapy itself can selectively enrich CSCs, leading to the expansion of this resistant cell population and subsequent tumor relapse. Investigating the significance of cancer stem cells in chemotherapy resistance offers valuable insights into the underlying mechanisms driving this resistance and helps researchers identify specific molecular pathways and signaling networks that contribute to the survival and drug resistance of these cells. By understanding these mechanisms, novel therapeutic targets can be identified and targeted therapies can be developed to overcome resistance.

Cancer organoid models originating from multiple types of tumors, including urothelial carcinoma, have been generated to validate the effectiveness of candidate therapeutic treatments [60, 61]. These three-dimensional, patient-derived cultures faithfully recapitulate the genetic, molecular, and histopathological characteristics of primary tumors, making them an ideal platform for investigating tumor heterogeneity, treatment response, and resistance mechanisms. In this study, we established patient-derived urothelial carcinoma (UC) organoids to gain deeper insights into the pathophysiology of chemotherapy resistance in bladder cancer. Notably, our findings revealed that the proportion of cancer stem cells (CSCs) was significantly elevated in chemotherapy-resistant UC organoids. Moreover, chemotherapy itself further reinforced the stemness phenotype, as evidenced by the elevated expression of key stemness markers, including CD44, ALDH1, and SOX2. These results strongly reinforce the pivotal role of CSCs in sustaining tumor persistence, driving disease recurrence, and conferring resistance to therapy, highlighting CSCs as a crucial therapeutic target in overcoming chemoresistance in bladder cancer. Long non-coding RNAs (lncRNAs) are critical epigenetic regulators involved in a wide range of cellular processes, including stem cell pluripotency, tumor progression, and chemotherapy resistance. Emerging evidence suggests that lncRNAs play a pivotal role in modulating the molecular networks that drive drug resistance, making them promising therapeutic targets for overcoming treatment failure in bladder cancer. In this study, we employed RNA sequencing (RNA-seq) to systematically screen for key regulatory genes associated with chemoresistance, providing a comprehensive molecular landscape of resistant tumor cells. Our analysis revealed a marked upregulation of multiple tumor-associated lncRNAs, including LUCAT1, in GEM-resistant bladder cancer organoids, highlighting their potential role in chemoresistance mechanisms. Notably, emerging evidence has demonstrated that LUCAT1 plays crucial roles in both embryonic stem cell differentiation and tumor progression. Consistently, our findings demonstrated that LUCAT1 expression was significantly elevated in GEM-resistant BC cells, and LUCAT1 promotes chemoresistance in bladder cancer cells by enhancing their stemness.

Interestingly, co-culture with GEM-resistant BC cells enhances both the stemness phenotype and chemoresistance of BC cells. Given that exosomes are essential messengers in cell-to-cell communication and recent studies have suggested that exosomes originating from CSCs facilitate drug resistance by transferring miRNAs, we hypothesized that exosomes derived from BCSCs may transfer LUCAT1, thereby facilitating the horizontal transfer of drug-resistant factors. Exosomes, recently identified as key communicators in intercellular signaling, play a crucial role in regulating tumor immune responses, chemoresistance, angiogenesis, and metastasis. For example, exosomes derived from BC cells can be internalized by human lymphatic endothelial cells (HLECs) and epigenetically regulated genes expression, ultimately resulting in lymphangiogenesis and lymphatic metastasis. Recent studies have also provided evidence that CSC-derived exosomes can transfer drug-resistant traits horizontally by transmitting proteins, DNA, miRNAs and lncRNAs. Furthermore, this study revealed that LUCAT1 was up-regulated in urinary exosomes from GEM-resistant patients, and exosomal LUCAT1 levels were negatively correlated with chemotherapy response in these patients. Moreover, exosomes originating from GEM-resistant BC cells were capable of delivering LUCAT1, facilitating the transfer of drug-resistant characteristics from GEM-sensitive to GEM-resistant cells. In vivo, exosome-mediated transmission of LUCAT1 promoted GEM chemoresistance, underscoring its critical role in enhancing both the stemness phenotype and chemoresistance. Further investigation into LUCAT1-mediated regulatory networks could pave the way for novel therapeutic strategies aimed at targeting resistant tumor cell populations and improving treatment efficacy in bladder cancer.

Mechanistically, our comprehensive transcriptional analysis revealed that LUCAT1 significantly upregulates HMGA1 expression, a key regulatory gene associated with cancer stem cell properties. Moreover, the suppression of the malignant phenotype in bladder cancer (BC) cells induced by LUCAT1 silencing was reversed upon HMGA1 overexpression, highlighting its functional relevance. LncRNAs regulate various biological processes through multiple mechanisms, including epigenetic modifications, transcriptional regulation, and acting as competitive endogenous RNAs. Fluorescence in situ hybridization analysis demonstrated that LUCAT1 is predominantly localized in the cytoplasm, suggesting a potential role in post-transcriptional gene regulation. In support of this hypothesis, our comprehensive sequence analysis and subsequent experimental validation demonstrated that LUCAT1 interacts with IGF2BP2, an mRNA-binding protein that stabilizes HMGA1, and enhances HMGA1 mRNA stability through an m6A-dependent mechanism. However, the precise molecular interactions between LUCAT1 and IGF2BP2, along with the exact binding site locations, remain to be elucidated. Further in-depth investigations are required to fully decipher the complexities of this regulatory network. Additionally, developing safe and effective interventions targeting the LUCAT1/IGF2BP2/HMGA1 signaling axis to effectively inhibit the stemness transformation of bladder cancer cells and suppress chemotherapy resistance warrants further investigation.

## Conclusions

Our investigation has revealed that cancer stem cell-derived exosomal LUCAT1 enhances the stemness phenotype and chemoresistance of bladder cancer cells by upregulating HMGA1 expression through its interaction with IGF2BP2, thereby contributing to the oncogenicity of bladder cancer. These findings provide new insights into the mechanisms underlying chemoresistance in bladder cancer. Collectively, our study highlights the clinical relevance of LUCAT1 as a potential tumor biomarker and underscores its promise as both a diagnostic and therapeutic target for bladder cancer.

## Electronic supplementary material

Below is the link to the electronic supplementary material.

Supplementary Material 1

Supplementary Material 2

Supplementary Material 3

Supplementary Material 4

## Data Availability

The data generated and analyzed during this study are included in the article or can be accessed upon request from the corresponding authors.
