# Supplementary material for: Exosome-transmitted LUCAT1 promotes stemness transformation and chemoresistance in bladder cancer by binding to IGF2BP2
Source: J Exp Clin Cancer Res. 2025 Mar 3;44:80. doi: 10.1186/s13046-025-03330-w (PMC11874664; doi:10.1186/s13046-025-03330-w)
Supplement: Supplementary file 1 — Supplementary Material 1 [file 13046_2025_3330_MOESM1_ESM.docx]

Supplemental Materials and Methods

Clinical sample collection and cell culture

Fresh bladder cancer tissue samples and pair-matched normal tissue samples were obtained from patients who underwent radical cystectomy. After resection fresh bladder cancer tissue and pair-matched normal adjacent bladder tissue obtained from the same patient were snap-frozen in liquid nitrogen immediately. All patients included in this study signed informed consent and this study was approved by the Institutional Review Board of Zheng Zhou University First Hospital and The First Affiliated Hospital of Shenzhen University, China. The normal urothelial cell line and bladder cancer cell lines were purchased from the Institute of Cell Research, Chinese Academy of Sciences, Shanghai, China. Corresponding plates were placed at 37 °C with a humidified atmosphere of 5 % CO_2_ in incubator.

RNA extraction and quantitative real-time PCR

The total RNA of the tissue samples and the transfected cells were extracted using the Trizol reagent (Invitrogen, Carlsbad, CA, USA) according to the manufacturer’s instructions. The detailed primer sequences included in this study are shown in Supplementary Table2. Quantitative real-time PCR was performed using the ABI PRISM 7000 Fluorescent Quantitative PCR System (Applied Biosystems, Foster City, CA, USA) according to the manufacturer’s instructions and normalized to β-actin or U6 small nuclear RNA.

Flow cytometry analysis assay

BCSCs were isolated from bladder cancer cells (BCCs) using flow cytometry based on the stem cell markers ALDH1. The BCSCs were resuspended in DMEM/F-12 supplemented with 20 ng/mL EGF, 20 ng/mL bFGF and 2% B27 and then cultured in RPMI 1640 supplemented with 10% FBS. The cells were incubated at 37°C in a humidified 5% CO2 atmosphere. Cell apoptosis was determined by flow cytometry. Briefly, cells were cultured in normal medium and transfected with the corresponding shRNA. Cells were collected after transfection for 48 h. Cell apoptosis was determined by PE Annexin V apoptosis detection kits (BD Pharmingen, San Diego, CA, USA). Finally, cell apoptosis was determined using flow cytometry (EPICS, XL-4, Beckman, CA, USA). Experiments were repeated at least three times.

**Cell self-renewal assays**

BC cells were collected after transfection with the corresponding vector for 48 h; then, 2×10^2^ BC cells were seeded on an ultra-low attachment surface 24-well plate (Corning, USA). BC cells were resuspended in DMEM supplemented with 10% FBS and incubated for 7 d at 37°C. Finally, the spheres were visualized under an optical microscope (Olympus, Japan) and a confocal laser-scanning microscope (Leica, Germany). For single-cell spheroid-formation assay, BC cells were seeded on an ultra-low attachment surface 96-well plate (Corning, USA). BC cells were resuspended in DMEM supplemented with 10% FBS and incubated for 7 d at 37°C. Finally, the spheres were visualized under a confocal laser-scanning microscope (Leica, Germany).

Western blotting analysis

Total cell lysates were prepared in a 1× sodium dodecyl sulfate buffer. Total protein was separated by sodium dodecyl sulfate-polyacrylamide gel electrophoresis and transferred onto nitrocellulose membranes. Then the membrane was blocked with 5% non-fat milk and incubated with primary antibodies at 4°C overnight. After incubation with the primary antibody IGF2BP2 (1:2000; Abcam, Hong Kong, China), the blots were incubated with goat anti-rabbit secondary antibody (Abcam, Hong Kong, China) and visualized with enhanced chemiluminescence using an ECL kit (Beyotime Biotechnology, China).

FISH, immunohistochemistry and immunostaining

RNA fluorescent in situ hybridization (FISH), immunohistochemistry and immunostaining were performed following standard protocols as previously reported[49]. LUCAT1 and U6 probes were designed and synthesized by Ribobio Company and labeled with Cy3 fluorescent dye. Fluorescence detection was performed with a confocal laser-scanning microscope (Leica, Germany). The primary antibody HMGA1/CD44/Ki67/CK7/UPII were purchased from Abcam, Hong Kong, China. The primary antibody SOX2 and fluorescence secondary antibody were purchased from Cell Signaling Technology, USA. Fluorescence detection was performed with a fluorescence microscopy.

Mouse model experiments

All animal experiments were approved by the Institutional Animal Care and Use Committee (IACUC) of The First Affiliated Hospital of Zhengzhou University and The First Affiliated Hospital of Shenzhen University and conducted in accordance with its recommendations and ethical regulations. For the tumour xenograft implantation experiment, UC organoids were inoculated subcutaneously into 5-week-old male BALB/c nude mice (Vital River, Beijing, China), which were subsequently sacrificed 5 weeks later. For the tumor-initiating capacity assay, the indicated cells mixed with an equal volume of Matrigel were injected into BALB/c nude mice and the tumor formation was observed 2 months later. BC cells ratios were obtained using extreme limiting dilution analysis (https://bioinf.wehi.edu.au/software/elda/). Five BALB/c nude mice were sampled at each time point for the tumor initiation assays. For the cell fluorescence trace system assay, BC cells labeled with green and red fluorescence were mixed in equal proportions and were inoculated subcutaneously into 5-week-old male BALB/c nude mice. Two weeks after tumor cell inoculation, the nude mice were selected randomly for treatment with chemotherapy or PBS control, administered by intraperitoneal injection. After one GEM cycle, the frozen section of xenografts was visualized using fluorescence microscopy. For the in vivo chemotherapy assay, xenografts were established on the left side of the dorsum and five mice were used. Two weeks after tumor cell inoculation, the nude mice were selected randomly for treatment with corresponding exosome, administered by intratumoral injection. Then, the corresponding exosome treated nude mice were further selected randomly for treatment with chemotherapy or PBS control, administered by intraperitoneal injection. For one GEM cycle, five consecutive treatments of gemcitabine (40 mg/kg) were applied on days 1, 4, 7, 10 and 14, followed by a 7-day interval. After two GEM cycles, xenografts were collected for measurement analysis.
