# Supplementary material for: Exosome-transmitted LUCAT1 promotes stemness transformation and chemoresistance in bladder cancer by binding to IGF2BP2
Source: J Exp Clin Cancer Res. 2025 Mar 3;44:80. doi: 10.1186/s13046-025-03330-w (PMC11874664; doi:10.1186/s13046-025-03330-w)
Supplement: Supplementary file 2 — Supplementary Material 2 [file 13046_2025_3330_MOESM2_ESM.docx]

**Supplementary Table1. Correlation between LUCAT1 expression and clinicopathological features of BC patients.**

| **Parameters Total** | **Group** | **Total** | **LUCAT1 expression** | | ***p* value** |
| --- | --- | --- | --- | --- | --- |
|  |  |  | **High** | **Low** |  |
| Gender | Male | 79(75%) | 58(55%) | 21(20%) | *p*>0.05 |
|  | Female | 27(25%) | 20(18%) | 7(7%) |  |
| Age (years) | < 60 | 37(35%) | 27(26%) | 10(9%) | *p*>0.05 |
|  | ≥ 60 | 69(65%) | 51(48%) | 18(17%) |  |
| Tumor size (cm) | < 3 cm | 42(40%) | 26(25%) | 16(15%) | *p*<0.05 * |
|  | ≥ 3 cm | 64(60%) | 52(48%) | 12(12%) |  |
| Multiplicity | Single | 59(56%) | 39(36%) | 20(20%) | *p*>0.05 |
|  | Multiple | 47(44%) | 39(36%) | 8(8%) |  |
| Histological grade | L | 48(45%) | 30(28%) | 18(17%) | *p*<0.05 * |
|  | H | 58(55%) | 48(45%) | 10(10%) |  |
| Tumor stage T | Ta,T1 | 26(25%) | 14(13%) | 12(12%) | *p*<0.01 ** |
|  | T2-T4 | 80(75%) | 64(60%) | 16(15%) |  |
| Lymph nodes metastasis | NO | 92(87%) | 66(62%) | 26(25%) | *p*>0.05 |
|  | YES | 14(13%) | 12(11%) | 2(2%) |  |

**p*<0.05; ***p*<0.01; *p*<0.05 was considered significant (Chi-square test between 2 groups).

**Supplementary Table2. The primer sequences included in this study.**

| **Gene** | **Accession number** | **primer sequences (5’-3’)** | |
| --- | --- | --- | --- |
| SOX2 | HGNC: 11195 | Forward | AGGACTGAGAGAAAGAAGAGGAG |
|  |  | Reverse | CGCCGCCGATGATTGTTAT |
| CD44 | HGNC: 1681 | Forward | CTGCCGCTTTGCAGGTGTA |
|  |  | Reverse | CATTGTGGGCAAGGTGCTATT |
| ALDH1 | HGNC: 402 | Forward | CCGTGGCGTACTATGGATGC |
|  |  | Reverse | GCAGCAGACGATCTCTTTCGAT |
| LUCAT1 | HGNC: 48498 | Forward | TTGGCACCAGAGACCACAAA |
|  |  | Reverse | GGGCGACAGAGCGAAACTCT |
| HMGA2 | HGNC: 5009 | Forward | ACCCAGGGGAAGACCCAAA |
|  |  | Reverse | CCTCTTGGCCGTTTTTCTCCA |
| HMGA1 | HGNC: 5010 | Forward | AGCGAAGTGCCAACACCTAAG |
|  |  | Reverse | TGGTGGTTTTCCGGGTCTTG |
| IGF2BP2 | HGNC: 28867 | Forward | ACACAGACACAGAAACCGCC |
|  |  | Reverse | AACTGATGCCCGCTTAGCTT |
| METTL3 | HGNC: 17563 | Forward | CAAGCTGCACTTCAGACGAA |
|  |  | Reverse | GCTTGGCGTGTGGTCTTT |
| β-actin | HGNC: 132 | Forward | GCGGACTATGACTTAGTTGCGTTACA |
|  |  | Reverse | TGCTGTCACCTTCACCGTTCCA |
